# Closing the Gap: Membrane Contact Sites in the Regulation of Autophagy

**DOI:** 10.3390/cells9051184

**Published:** 2020-05-09

**Authors:** Verena Kohler, Andreas Aufschnaiter, Sabrina Büttner

**Affiliations:** 1 Department of Molecular Biosciences, The Wenner-Gren Institute, Stockholm University, 106 91 Stockholm, Sweden; verena.kohler@su.se; 2 Department of Biochemistry and Biophysics, Stockholm University, 106 91 Stockholm, Sweden; andreas.aufschnaiter@dbb.su.se; 3 Institute of Molecular Biosciences, University of Graz, 8010 Graz, Austria

**Keywords:** autophagy, ER–mitochondria encounter structure, ERMES, lipophagy, membrane contact sites, mitochondria-associated membranes, MAMs, mitophagy, nucleus–vacuole junction, pexophagy, piecemeal microautophagy of the nucleus

## Abstract

In all eukaryotic cells, intracellular organization and spatial separation of incompatible biochemical processes is established by individual cellular subcompartments in form of membrane-bound organelles. Virtually all of these organelles are physically connected via membrane contact sites (MCS), allowing interorganellar communication and a functional integration of cellular processes. These MCS coordinate the exchange of diverse metabolites and serve as hubs for lipid synthesis and trafficking. While this of course indirectly impacts on a plethora of biological functions, including autophagy, accumulating evidence shows that MCS can also directly regulate autophagic processes. Here, we focus on the nexus between interorganellar contacts and autophagy in yeast and mammalian cells, highlighting similarities and differences. We discuss MCS connecting the ER to mitochondria or the plasma membrane, crucial for early steps of both selective and non-selective autophagy, the yeast-specific nuclear–vacuolar tethering system and its role in microautophagy, the emerging function of distinct autophagy-related proteins in organellar tethering as well as novel MCS transiently emanating from the growing phagophore and mature autophagosome.

## 1. Introduction

**Figure 1:**
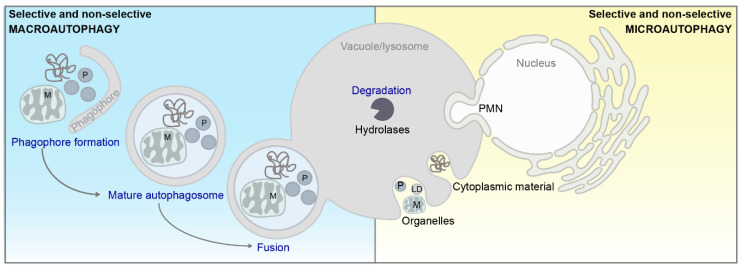
Schematic overview of macro or bulk- (blue)- and microautophagy (yellow). For a general description of autophagy, please see main text. PMN = piecemeal microautophagy of the nucleus, P = peroxisome, M = mitochondria, LD = lipid droplet.

Intracellular compartmentalization in form of membrane-bound organelles represents a defining feature of eukaryotic cells. This spatial separation is a prerequisite for the generation of dedicated microenvironments to accommodate incompatible biochemical reactions. At the same time, efficient communication systems between individual organelles need to be in place to maintain cellular homeostasis, which is facilitated either via vesicular transport or direct contact between two organelles at so-called membrane contact sites (MCS) [1,2]. MCS have been described to govern essential cellular functions, including lipid metabolism and the transfer of small signaling molecules, including calcium ions (Ca^2+^) [3]. MCS can be classified as contacts between either identical (homotypic) or different (heterotypic) organelles [1]. According to recent guidelines, MCS are defined by (i) the presence of protein–protein or protein–lipid interactions that mediate tethering forces between the membranes, (ii) a lack of membrane fusion and fusion intermediates, (iii) a specific function of the contact, and (iv) a defined proteome and lipidome required for all previously mentioned definitions [1]. While MCS between virtually all organelles have been identified in recent years [4,5], a large number of heterotypic MCS involve the endoplasmic reticulum (ER) [6,7], reflecting the important role of the ER for cellular metabolism and homeostasis. The molecular architecture of distinct MCS seems to be (at least in part) evolutionary conserved, and the complex communication networks established via MCS contribute to the coordinated adaption of participating organelles to different environmental challenges across the eukaryotic kingdom [4]. Accumulating evidence indicates that MCS play a decisive role in regulating the breakdown of cellular material via autophagy in both yeast and higher eukaryotic organisms. While an intricate connection between autophagy and distinct processes governed by MCS—such as Ca^2+^ signaling and lipid metabolism—is well established [8,9,10,11], recent studies suggest a more direct involvement of MCS in the regulation of autophagy. Indeed, all major subtypes of autophagy, including macro- and microautophagy (Figure 1) as well as respective cargo-selective and non-selective (bulk) autophagic subforms, have been to some extend connected to MCS [12,13,14,15,16]. As autophagy is generally described as a cytoprotective mechanism that mediates cellular health and longevity, and its dysregulation is associated with a wide range of human diseases [17], the emerging interplay between autophagy and MCS might have important physiological as well as pathophysiological implications. While we intend to give a brief overview of selected aspects of autophagy that we touch upon in later sections, a comprehensive description of this multi-faceted process can be found in several excellent reviews (e.g., [12,18,19,20,21,22,23,24]).

The core molecular machinery governing autophagy is highly conserved from simple single-celled eukaryotes like the baker’s yeast *Saccharomyces cerevisiae* up to complex multicellular organisms like humans [13,25]. During macroautophagy, cargo is sequestered into a double-membraned vesicle termed autophagosome, which subsequently fuses with the lysosome or the yeast counterpart, the vacuole [26]. This involves a series of tightly-regulated steps, including initiation of the so-called phagophore (or isolation membrane), its elongation with lipid resources and autophagosome formation, fusion with the lysosome/vacuole, degradation of the cargo by soluble hydrolases, and finally recycling of macromolecules (Figure 1). The lipid resources required for phagophore initiation/elongation are suggested to originate from different organelles [13,22,23]. During selective forms of macroautophagy, adaptor proteins interact with components of the core machinery of autophagy as well as with a series of receptors to target the respective organelle to the expanding phagophore membrane ([22,27]; Figure 1). Microautophagy refers to the direct engulfment of cargo into the vacuole/lysosome via membrane invaginations and scissions, resulting in intravacuolar vesicles that are subsequently degraded [14,15,16]. Selective microautophagy has been described for the turnover of mitochondria [28], peroxisomes [29], parts of the nucleus [30,31,32,33], lipid droplets [34,35], the ER [36], and the cytoplasm [37]. In this review, we focus on the emerging nexus between MCS and various subtypes of autophagy in both baker’s yeast and mammals, highlighting similarities and differences between these species.

## 2. Membrane Contact Sites at a Glimpse

**Figure 2:**
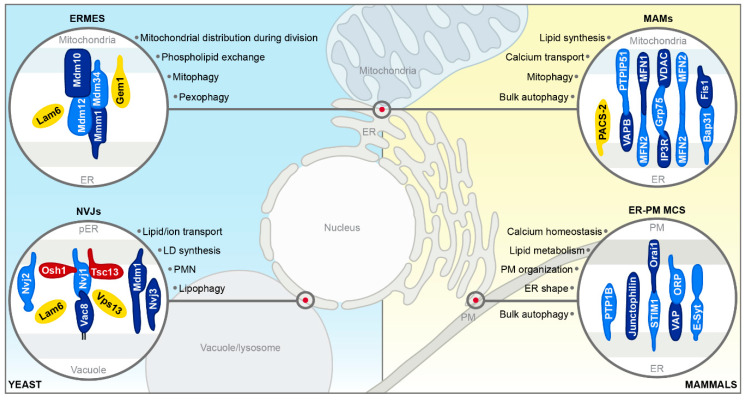
Schematic overview of selected membrane contact sites. Tether proteins (blue), proteins with other functions (red) and regulatory proteins (yellow) are depicted. For a general description of membrane contact sites (MCS) in yeast (blue) and mammals (yellow), please see main text. ER = endoplasmic reticulum; ERMES = ER–mitochondria encounter structure; pER = perinuclear ER; NVJs = nucleus–vacuole junctions; LD = lipid droplet; PMN = piecemeal microautophagy of the nucleus; MAMs = mitochondria-associated membranes; PM = plasma membrane.

Interorganellar communication routes are crucial to ensure immaculate function of a cell, and dedicated tethering machineries at MCS facilitate physical connections between organelles [1]. Two bi- or monolayer membrane-bound organelles that are connected by MCS are closely apposed, with distances mostly in the range of 10–30 nm, but do not fuse [1,38]. MCS rely on distinct protein- and lipid composition for efficient tethering and proper function [39,40,41,42,43,44]. As most MCS are composed of multiple tethering machines, abolishing organellar contact by deleting a single tethering pair is, with certain exceptions, usually not possible [1,44,45]. MCS are highly dynamic, changing in size, abundance and protein/lipid composition in response to various environmental and metabolic cues or forming only transiently to fulfil temporarily restricted functions [1,4]. The first contact sites described in yeast were the nucleus–vacuole junctions (NVJs), connecting the nucleus/perinuclear ER with the vacuole [45]. Studies on NVJs provided first evidence that MCS are not only hubs of metabolic exchange but in addition contribute to the regulation of autophagy [30,31]. To date, several other MCS emanating from the ER have been shown to not only facilitate lipid synthesis and trafficking, thereby indirectly impacting on diverse cellular processes including autophagy [46,47,48,49,50], but to also directly mediate distinct autophagic processes. This includes for instance the MCS between a growing phagophore and the ER during early stages of autophagy, allowing the direct transfer of lipid resources needed for membrane extension [51,52,53,54]. While contact sites between most organelles within a cell have been described (for a detailed review, please see [1,55]), we will focus only on those MCS implicated in the coordination of autophagic processes (Figure 2): The NVJs in yeast, the contacts between the ER and mitochondria in yeast and mammals, and the MCS connecting the ER and the plasma membrane in mammalian cells. We will provide a brief overview of the molecular architecture of these MCS in this section and discuss their contribution to autophagy as well as the different contacts established by the phagophore and the autophagosome in detail in Section 3.

### 2.1. ER–Mitochondria Encounter Structure in Yeast

The ER–mitochondria encounter structure (ERMES) is present as 2 to 10 distinct foci per cell and is crucial for interorganellar phospholipid exchange [56,57,58], mitochondrial distribution during cell division [59], and efficient mitophagy [60]. ERMES is formed by four core proteins: the ER membrane protein Mmm1, the two outer mitochondrial membrane proteins Mdm10 and Mdm34, and the cytoplasmic component Mdm12, which is required for structural integrity. Mutation or deletion of any of its components results in complete ERMES disappearance and respiratory deficiency ([56]; Figure 2). Gem1, a Ca^2+^-binding rho-like GTPase, acts as negative regulator of ERMES formation, affecting size and number of ERMES foci and influencing phospholipid homeostasis [61]. The sterol transporter Lam6 localizes to ERMES as well, driving contact expansion, and further governs the cross-regulation between ERMES and contact sites emanating from the vacuole to the perinuclear ER/nucleus and mitochondria [55,62,63,64]. Yeast harbors a second tethering system connecting the ER with mitochondria, formed by the interaction between the mitochondrial membrane protein Tom5 and the ER-membrane protein complex (EMC) that consists of 6 conserved proteins, Emc1-6, and governs among others ER-associated degradation (ERAD) [65]. So far, EMC-mediated contacts have not been shown to contribute to the coordination of autophagic processes, and EMC and ERMES facilitate organellar tethering independently of each other [66].

### 2.2. ER–Mitochondria Contact Sites in Mammals

The mammalian counterpart of yeast ER–mitochondria contact sites (ERMES and Tom5-EMC) are the mitochondria-associated membranes (MAMs), sometimes referred to as mitochondria–ER contact (MERC) [67,68]. While the tethering proteins connecting the ER and mitochondria differ between yeast and mammalian cells, regulators such as Lam6 and Gem1 are conserved [67]. Just as in yeast, MAMs represent highly dynamic platforms for the exchange of biochemical information and efficient lipid synthesis and are enriched in sphingolipids and cholesterol [46,69,70,71,72,73,74,75].

One MAM tethering machinery is composed of the Ca^2+^-dependent proteins inositol 1,4,5-triphosphate receptor (IP3R), the growth receptor protein 75 (Grp75) and the mitochondrial voltage-dependent anion channel (VDAC) [41]. Another tethering system consists of the outer mitochondrial membrane protein PTPIP51 and the vesicle associated membrane protein B (VAPB) [76]. Moreover, the mitochondrial fission factor Fis1 and the ER protein Bap31 can connect the ER with mitochondria at MAMs and, by interacting with procaspase 9, transfer apoptotic signals ([77]; Figure 2). All tethering complexes mentioned are involved in Ca^2+^ homeostasis at the ER-mitochondria interface. The sorting protein PACS-2 represents a crucial regulator of MAM formation, and its depletion leads to detachment of the ER from mitochondria [78]. In addition, the mitofusins MFN1 and MFN2, two GTPases, are reported to control MAM abundance and distribution. MFN2 decorates the complete outer mitochondrial membrane but is strongly enriched at MAMs and is suggested to transiently tether the ER to mitochondria [79], even though controversial findings exist (reviewed in [80,81]). Numerous additional proteins have been shown to localize to MAMs [39,78,82,83,84], and alterations in the protein composition at MAMs are implicated in several human diseases [81,85,86,87].

### 2.3. ER–Plasma Membrane Contact Sites in Mammals

The contacts between the ER and the plasma membrane are ubiquitous structures found in most cell types, though with strikingly different architecture, and have been visualized in high resolution in yeast and mammals [88,89]. As most MCS, these contacts play vital roles in Ca^2+^ homeostasis and lipid metabolism but are also regulators of ER shape and architecture as well as of plasma membrane organization [90,91,92]. Extended synaptogamins (E-Syts) are signature molecules for these contacts and regulate the distance between the plasma membrane and the ER in a Ca^2+^-dependent manner [89]. Upon Ca^2+^ depletion in the ER, the ER-resident stromal interaction molecule 1 (STIM1) binds and activates Orai1 Ca^2+^ channels at the plasma membrane, leading to Ca^2+^ influx [43,49].

VAMP (vesicle associated membrane protein)-associated proteins (VAPs) are highly conserved ER-resident proteins that interact with a wide range of FFAT-motif containing proteins, among them many tethers that connect the ER and other organelles [93,94]. Oxysterol-binding protein-related proteins (ORPs) and other FFAT-containing proteins mediate ER-plasma membrane tethering via interaction with VAPs or phosphaditylinositol-4-phosphate [5,95]. VAP proteins interacting with distinct lipid transport proteins (e.g., CERT, OSBP1, ORP9, or ORP10) have been recently described as molecular determinants of MCS between the ER and the trans-Golgi network in mammalian cells [94]. Furthermore, ER-resident PTP1B as well as Junctophilins were described to play a role as tethering components ([96,97]; Figure 2). In yeast, three conserved protein families were identified as ER–plasma membrane tethers. Among them are the tricalbins Tcb1-3 (homologous to E-Syt1-3), which together with Scs2/22 (the mammalian VAP counterparts), and Ist2 (related to mammalian TMEM16 ion channels) physically connect the ER with the plasma membrane, ensuring proper lipid synthesis and maintenance of ER physiology [48,98,99,100,101].

### 2.4. Nucleus–Vacuole Junctions in Yeast

NVJs enable the contact between the vacuole and the perinuclear ER and consist of one main tethering unit, the integral ER protein Nvj1 and the highly palmitoylated vacuolar protein Vac8 [45]. NVJs govern lipid and ion transport and function as sites for lipid droplet (LD) synthesis under stress conditions [102]. Moreover, these contact sites are essential for piecemeal microautophagy of the nucleus (PMN), a specialized form of selective autophagy [31,103,104]. NVJs expand upon entry into stationary phase, distinct dietary restrictions, and treatment with different stressors, including ER stress [45,105]. Accessory proteins such as Nvj2, Nvj3 and Mdm1 serve as regulators of NVJs under distinct conditions and concentrate at these contact sites to adapt abundance, distribution and function to respective cellular needs ([44,106]; Figure 2). Nvj1 links NVJs physically and functionally to lipid metabolism via direct interaction with the ER-resident enoyl reductase Tsc13, an essential enzyme of the very long-chain fatty acid elongation cycle, as well as the sterol transporter Osh1, a protein homologous to mammalian oxysterol-binding proteins [107,108,109,110]. NVJs establish a microenvironment that is enriched in the vacuolar transporter chaperone (VTC) complex, while it completely excludes both the V-ATPase and the nuclear pore complex [45,104]. Other proteins, including Lam6 and Vps13, have been shown to transiently localize to NVJs depending on the growth conditions, but to be present at other MCS as well [111,112]. Interestingly, the direct mammalian counterpart of NVJs has not been discovered yet, even though MCS between the ER and endosomes have been described and characterized [113,114].

## 3. The Nexus between Membrane Contact Sites and Autophagy

Accumulating evidence reveals a regulatory role of MCS in selective and non-selective forms of autophagy. In particular, the contact sites emanating from the ER contribute to various steps of autophagy, ranging from autophagosome biogenesis at ER-plasma membrane or ER–mitochondria contacts to the role of the latter in the autophagic turnover of mitochondria, namely mitophagy. The identification of contact sites between the growing phagophore and the ER established by distinct autophagy-related (Atg) proteins, a role for MAMs and their associated proteins in autophagosome biogenesis, as well as interactions between crucial regulators of autophagy and MCS tether proteins highlight the importance of organellar connectivity in form of physical contact for autophagy.

### 3.1. Autophagy en Passant

**Figure 3:**
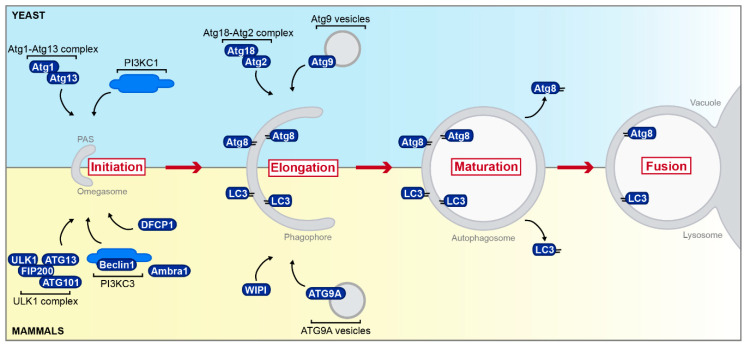
Simplified overview of autophagy in yeast and mammals. Selected aspects of the autophagic process from initiation at the phagophore assembly site (PAS) in yeast (blue) or the omegasome in mammals (yellow) to fusion with the vacuole/lysosome including proteins mentioned in later sections are depicted. Please see main text for further details. PAS = phagophore assembly site.

Initiation of macroautophagy takes place at a perivacuolar punctate structure termed phagophore assembly site (PAS) in yeast [115], while the omegasome, an omega-shaped structure in close proximity to the ER, is proposed as the origin of autophagosomes in mammalian cells ([23,116]; Figure 3). Atg proteins involved in the initiating steps at the PAS in yeast include the Atg1-Atg13 complex as well as the phosphoinositide 3-kinase complex I (PI3KC1) [117]. The mammalian ULK1 complex is the counterpart of the yeast Atg1 complex and consists of ULK1 (orthologous to yeast Atg1), ATG13 (homolog of yeast Atg13), FIP200, and ATG101 [23,118]. At the ER, the ULK1 complex activates the class III phosphatidylinositol 3-kinase complex (PI3KC3; with Beclin1 as one constituent and Ambra1 as its regulatory adapter). Subsequent production of phosphatidylinositol 3-phosphate (PI3P) recruits the double FYVE-containing protein 1 (DFCP1) and promotes the formation of the omegasome [116,119]. Atg9, the only transmembrane protein of the yeast autophagy machinery known so far [120], is synthesized in the ER, sorted into small vesicles that serve as membrane source during the early steps of autophagosome formation [121], and associates with the Atg18-Atg2 complex [12]. Similarly, its mammalian counterpart ATG9A coordinates membrane transport from several donor sources to the omegasome [122]. Interaction of ATG9A with one of the WIPI (WD-repeat protein interacting with phosphoinositides) proteins [123], the mammalian homologs of yeast Atg18, is important for vesicle movement and membrane delivery [23]. Yeast Atg8 and its mammalian homolog LC3 and isoforms thereof are attached to the growing autophagosomal membrane via covalent attachment to phosphatidylethanolamine, a processes that requires a dedicated ubiquitin-like conjugation system [23,124,125,126,127,128,129]. This system, composed of Atg5-Atg12-Atg16 (also called ATG16L complex in mammalian cells), decorates pre-phagophore structures and phagophores but dissociates from mature autophagosomes. Furthermore, this complex dictates the site of LC3/Atg8 lipidation [130,131]. While Atg8 and its mammalian counterparts are initially located on both membranes of the growing phagophore, they are deconjugated on the external membrane upon autophagosome maturation, allowing further rounds of conjugation [12,132]. Disassembly of the Atg machinery from the autophagosomal surface is a prerequisite for fusion with the vacuole/lysosome [133,134]. In yeast, the outer autophagosomal membrane fuses with the limiting membrane of the vacuole to release the inner vesicle as autophagic body into the vacuolar lumen [135], while mammalian autophagosomes can either form so-called amphisomes by fusion with late endosomes [136,137] or can directly fuse with lysosomes, forming autolysosomes. In addition, mammalian autophagosomes can deliver their content in a “kiss-and-run fusion”, which seems to be more common [138]. In particular, the early steps of autophagy—including phagophore expansion, autophagosome formation, and positioning—have been shown to depend on the efficient formation of MCS.

### 3.2. Membrane Contact Sites with the Growing Phagophore

**Figure 4:**
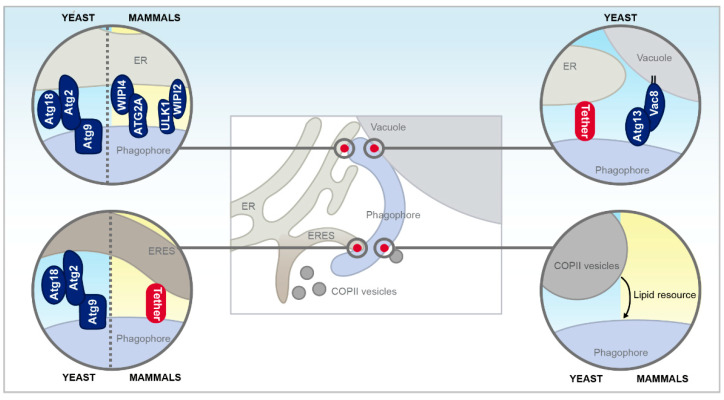
Membrane contact sites and functional interorganellar interaction with the growing phagophore in yeast and mammals. Besides direct membrane contacts sites between the vacuole, the endoplasmic reticulum (ER) and the phagophore, COPII vesicular transport between the ER exit sites (ERES), and the phagophore mediate autophagosome formation and elongation. Please see main text for further details.

The expanding phagophore itself has been shown to establish direct contact to the ER by specific protein tethers in both yeast and mammalian cells (Figure 4). These contacts are suggested to facilitate direct lipid transfer from the ER to support the growth of autophagosomes, thus representing an alternative to the established lipid delivery route via vesicle transfer. This involves, for instance, COPII vesicles in yeast and mammals and the ER-Golgi intermediate compartment (ERGIC) in mammals [51,139,140,141,142]. Additionally, the plasma membrane was shown to directly contribute to the formation of ATG16L-positive autophagosome precursors, thus most likely also serving as membrane reservoir [143].

Contact formation between the tip of the growing phagophore and the ER in yeast has been shown to be facilitated by a trimeric complex consisting of Atg9, Atg2, and Atg18 and to be essential for efficient autophagosome biogenesis [144]. Atg9 associated independently of Atg2 with the PAS and the extremity of the phagophore, and an interaction with Atg9 facilitated subsequent accurate targeting of Atg2 selectively to the phagophore extremities. Interestingly, compromising the Atg9-Atg2 interaction resulted in Atg2 spreading over the entire phagophore surface, leading to extensive and most probably non-functional tethering to the ER, possibly caused by the intrinsic ability of Atg2 to bind to PI3P. The interaction between Atg9 and Atg2 was not only prerequisite for phagophore growth but also for the subsequent recruitment of Atg18 [144]. Disruption of the ER-phagophore contacts established by Atg9-Atg2-Atg18 interaction severely compromised autophagy, most probably due to insufficient transfer of lipid resources from the ER to the growing phagophore [144]. Similarly, mammalian ATG2 was recently shown to adopt a rod-shaped conformation that enabled it to bind and bridge membranes in vitro both in its apo-form or complexed with WIPI4 (one of the mammalian Atg18 homologs), and thus might act as a tethering factor for autophagy [145,146]. The purified ATG2A-WIPI4 complex could bridge distances between 10 nm and 30 nm, typically found at MCS, and was proposed to tether the ER and the phagophore in vivo, just as its yeast counterpart [146]. In addition, phagophore extension was observed to require ATG2 targeting to ER-phagophore junctions enriched in PI3P. The tips of the rod-shaped ATG2 bound to neighboring vesicles, and additional binding of WIPI4 to one of these tips enabled the ATG2A-WIPI4 complex to tether PI3P-enriched vesicles with PI3P-free vesicles, mediating ER-phagophore assembly [147]. Additional ER-phagophore tethering was shown to be facilitated by the interaction between WIPI2, another homolog of yeast Atg18, the ULK1 complex and PI3P [148].

Others observed the phagophore to be associated with so-called ER exit sites (ERES), a distinct portion of the ER, where COPII vesicles bud off, suggesting ERES as regions of autophagosome formation in both yeast and mammals [149,150]. Interestingly, starvation conditions led to re-localization of the COPII machinery to the ERGIC in mammalian cells, which was necessary for generating vesicles for LC3 lipidation and recruiting early Atg proteins [141,142].

Upon pharmacological induction of autophagy via rapamycin in yeast, one edge of the phagophore decorated with Atg2, Atg18, and Atg9 was found in immediate vicinity to ERES. Moreover, most Atg8-positive PAS, phagophores and mature autophagosomes localized close to ERES. Interestingly, this connection seems evolutionary conserved, as a mammalian ERES marker and LC3 also colocalized upon rapamycin treatment [150]. Atg1 function was unaffected in cells unable to form ERES, indicating that these sites impact autophagy downstream of the Atg1 kinase complex [150]. Intriguingly, the PAS localized proximal to both the vacuole and the ER and often bridged the distance between these two organelles. Thus, the vacuole-ER contact might be linked to initiation of autophagosome formation. As this spatial linkage between expanding phagophores, ERES and vacuoles were frequently observed, the presence of two rather elusive tethering machineries in yeast was proposed [150]. Recently, an interaction between Vac8 and Atg13 has been suggested to facilitate the physical contact between PAS and the vacuole. Interestingly, the authors described the PAS and associated Atg proteins as forming a lipid-like condensate that led to liquid–liquid phase separation [151].

In addition to MCS connecting the growing phagophore and the ER [40,152,153,154,155], recent studies indicate that also the late autophagosome establishes contact to the ER. These MCS have been shown to be required for proper transport and positioning of the autophagosome, particularly in high cholesterol conditions [156]. While autophagic vacuoles (a term including autophagosomes, amphisomes, and autolysosomes) were scattered throughout the cytoplasmic space upon cholesterol depletion, high cholesterol promoted their enrichment at distinct spots at the perinuclear ER [156]. This tethering to the ER was modulated by the Rab7-interacting lysosomal protein (RILP) and the cholesterol-sensing Rab7 effector ORP1L. While RILP regulated the transport of mature autophagosomes, ORP1L was targeted to amphisomes and autolysosomes and contacted ER-resident VAPA under low cholesterol conditions. Thus, cholesterol levels sensed via ORP1L regulated autophagosome positioning, thereby governing the last steps of autophagy [156].

### 3.3. ER–Mitochondria Contacts as Hubs for Autophagy

Physical contact between the ER and mitochondria established by MAMs in mammalian cells and ERMES in yeast is central for various autophagic processes, in particular for mitophagy. Both MAMs and ERMES govern the selective degradation of unused/damaged mitochondria and mark the sites of mitochondrial fission, a prerequisite for subsequent engulfment into an autophagosome and vacuolar/lysosomal breakdown.

Phosphorylation of the yeast mitophagy receptor Atg32 on the outer mitochondrial membrane triggers its interaction with the adaptor protein Atg11, targeting the mitochondria to the phagophore, where association with Atg8 allows phagophore expansion and engulfment into an autophagosome. In yeast, efficient mitophagy requires the presence of all four ERMES tethers, forming puncta at the ER–mitochondria interface that determine the sites for mitochondrial fission [60,157,158,159,160]. Notably, an artificial ER–mitochondria tether (chiMERA; [56]) restored mitophagy, indicating that indeed loss of the contact itself rather than any additional function of distinct ERMES subunits compromises mitophagy in these mutants [60]. In addition, ERMES seems to be required for phagophore expansion, most probably supplying the growing phagophore with lipids from the ER [60,157], and mitophagosomes were observed to originate from these sites. Furthermore, Atg8 was found to physically interact with the ER-resident ERMES component Mmm1 in vivo [60,157]. Interestingly, efficient mitophagy further required mono-ubiquitinylation of the ERMES components Mdm34 and Mdm12 by the E3 ligase Rsp5, which was triggered by mitochondrial fission as a prerequisite of mitophagy [158]. This modification of Mdm34 was necessary for its colocalization with Atg32, Atg8, and Atg9. The authors speculate that Rsp5 might fulfil a similar function as Parkin in mammals [161] and that ubiquitinylated Mdm34 might act as an Atg32-independent mitophagy receptor to recruit Atg8 [158]. In addition to the role of ERMES in yeast mitophagy, this MCS was recently shown to be crucial for pexophagy as well [162]. Peroxisomes destined for degradation co-localized with ERMES, and loss of ERMES impaired pexophagy. A mutated Mdm34 variant with impaired ERMES tethering function also displayed reduced association with Pex11, leading to defects in pexophagy. Targeting of Vps1, a GTPase mediating peroxisomal fission [163,164], to peroxisomes at ER–mitochondria contacts required Atg11 and the pexophagy receptor Atg36 [164].

While a function for the mammalian ER–mitochondria contacts in pexophagy remains to be elucidated, their crucial role in mitophagy is well established. In mammalian cells, different mitophagy pathways exist, one of them being executed via PINK1 and Parkin. Upon mitochondrial depolarization, the mitochondrial kinase PINK1 phosphorylates several targets at the outer mitochondrial membrane, which recruits the E3 ubiquitin ligase Parkin, leading to increased ubiquitylation and recognition by autophagy receptors [165,166,167]. Interaction between autophagy receptors and LC3 attaches the targeted mitochondria to the expanding phagophore, thus promoting their sequestration into autophagosomes [168]. Following mitophagic stimuli, autophagosomes formed specifically at MAMs, and both PINK1 and the PI3PKC3 component Beclin1 localized at these sites, facilitating contact enlargement and formation of the omegasome [169]. Though already present at MAMs at basal levels, induction of mitophagy enforced the targeting of PINK1 and Beclin1 to these contact sites, and silencing of Beclin1 impaired mitochondrial clearance [169]. Moreover, MAMs have been shown to prevent apoptotic cell death by facilitating the efficient autophagic degradation of damaged mitochondria [170]. Mitochondrial dysfunction upon treatment with oxidized lipids triggered increased MAM abundance, accumulation of Beclin1 at these contact sites and a tight association of Beclin1 with PACS-2 and Grp75 [170]. The interaction between Beclin1 and PACS-2 not only promoted interorganellar tethering between ER and mitochondria but also initiated mitophagosome formation, and dysfunctional mitochondria were suggested to be sensed by MAMs [170].

Besides their contribution to mitophagy, MAMs have also been shown to impact bulk autophagy in various ways, recruiting autophagy-relevant proteins and serving as a hotspot for membrane resources [40,171,172]. Specific lipid rafts detected in MAMs have been shown to contribute to autophagosome formation, and the glycosphingolipid GD3 accumulated at these MAM-associated lipid rafts upon autophagy induction. A decrease in GD3 levels did not only alter MAM composition but also impaired autophagy [40]. Also the stimulation of autophagy via starvation required efficient MAM formation and triggered the relocalization of the PI3KC3 complex to MAMs, driven by an interaction of the PI3KC3 component ATG14 with the ER-resident SNARE protein syntaxin 17 [173]. MAM disruption via depletion of the tethers MFN2 or PACS-2 inhibited formation of LC3 puncta and impaired autophagosome formation [155]. Moreover, the PI3P-binding autophagy regulator DFCP1 has been suggested to contribute to the connection between the ER and mitochondria in response to starvation [173]. In sum, MAMs are not only essential for the coordination of mitophagy, but also contribute to early steps of bulk autophagy.

### 3.4. VMP1, the ‘Jack of All Trades’ in Mammalian Autophagy

Vacuole membrane protein 1 (VMP1), an ER-resident protein with no identified homolog in yeast [174,175,176], is an important regulator of autophagy in mammalian cells and impacts interorganellar connectivity and autophagosome formation at various stages [177,178]. VMP1 puncta were associated with the ER, mitochondria, peroxisomes, endosomes, and lipid droplets (LDs), with a prominent enrichment at the contacts sites between the ER and other organelles. Interestingly, loss of VMP1 increased MAM size and abundance, indicating that this protein negatively regulates the contact between ER and mitochondria [154]. VMP1 has also been shown to facilitate the formation of ER-microdomains necessary to establish MCS at sites of phagophore formation, thereby governing omegasome morphogenesis [154]. During starvation, VMP1 colocalized with LC3 and the PIP3-binding protein DFCP1 and was essential for efficient autophagy induction. Here, loss of VMP1 resulted in abnormal omegasome morphology and high levels of lipidated LC3, indicating that at least parts of the autophagic machinery are recruited independently of VMP1 to the ER sites of phagophore formation [154]. In line with this, another study reported that depletion of VMP1 impaired autophagic flux downstream of LC3 lipidation. As a result, protein levels of LC3 and FIP200 in microsomes increased, and the dissociation of LC3-positive autophagic structures was compromised due to a robust association with the ER [148]. Furthermore, lack of VMP1 enhanced the formation of the PI3KC3 complex and increased PI3P levels at autophagosome formation sites on the ER. WIPI2 aggravated the effects of VMP1 loss on ER-phagophore contacts by interacting with ULK1/FIP200 and PI3P. Moreover, VMP1 was shown to control phagophore-ER contacts by modulating the activity of the sarco/endoplasmic reticulum Ca^2+^-ATPase (SERCA). SERCA was recruited to autophagosome formation sites, where it associated with ULK1, an interaction that was enforced upon VMP1 depletion. In turn, enhanced SERCA activity alleviated the autophagy defects of VMP1 depleted cells [148]. Similar to the hypertethering observed in cells devoid of VMP1, the pharmacological inhibition of SERCA resulted in a general increase of contact sites emanating from the ER, including contacts to the phagophore, LDs, mitochondria, and endosomes [154]. This enforced tethering was due to spatially-defined changes in Ca^2+^ concentrations, as SERCA activity was necessary to decrease local Ca^2+^ levels around MCS, thereby inducing their disassembly [148]. VMP1 was further shown to be a negative regulator of the interaction between distinct autophagy-specific proteins and the ER-resident VAP proteins [179]. Autophagy induction triggered the recruitment of VAPs to ER-phagophore contact sites, thus enhancing their association with WIPI2 and FIP200 and enforcing the formation of the WIPI2/FIP200 ER-phagophore tethering complex. A lack of VMP1 further strengthened this interaction [179].

Moreover, VMP1 has also been implicated in autophagosome biogenesis at ER-plasma membrane contact sites established by E-Syts [153]. High levels of E-Syts, either via overexpression or induced by starvation, enforced ER-plasma membrane tethering, and triggered the recruitment of VMP1, LC3-positive vesicles, and other autophagy-relevant proteins—including Beclin1, ATG14L, and WIPI2—to these contact sites [153]. In E-Syt deficient cells, ER-plasma membrane contacts and autophagic vesicles were reduced, while the maturation and transport of autophagosomes were unaffected. Hence, ER-plasma membrane contact sites might contribute to autophagosomal biogenesis, guaranteeing temporal/spatial regulation of PI3P synthesis, in particular upon nutrient starvation [153].

Overall, VMP1 acts as a negative regulator of several MCS emanating from the ER, including the contacts to mitochondria, the plasma membrane, the growing phagophore and autophagosomes, and loss of VMP1 function seems to compromise autophagic flux via hypertethering.

### 3.5. Nucleus–Vacuole Junctions in Yeast Selective Autophagy

A particularly well-studied connection between MCS and autophagy is the piecemeal microautophagy of the nucleus (PMN) in yeast, which takes place at the MCS between the nucleus and the vacuole, the NVJs. PMN targets parts of the perinuclear ER and the nucleus for degradation and can be microscopically divided into five morphologically distinct stages: (i) the formation of NVJs by their main tethering units Nvj1 and Vac8, (ii) invagination of a portion of the nucleus into the vacuolar lumen, (iii) the formation of a tear drop-like bleb, (iv) pinching off of a tri-lamellar vesicle, and (v) degradation in the vacuolar lumen by hydrolases [31,180]. The intricate connection between NVJs and this specific form of autophagy has been established soon after their discovery, and a disruption of NVJ formation via deletion of either Nvj1 or Vac8 compromised PMN [181]. Still, this selective microautophagy is not essential for cellular survival upon starvation, pointing towards the existence of redundant degradation pathways for nuclear cargo [31,103]. Several enzymes involved in lipid metabolism localize at and facilitate the formation of NVJs, and vice versa PMN contributes to their turnover. For instance, Osh1 localizes to PMN blebs and can be targeted for degradation via PMN. At the same time, the proper formation of nuclear PMN vesicles requires the overlapping activities of Osh1 and other members of the oxysterol-binding protein family, and PMN is completely absent in yeast cells devoid of Osh1-7 [109]. Upon nutrient limitation, the enoyl reductase Tsc13 is not only sequestered as cargo into PMN vesicles in a Nvj1-dependent manner, but also controls the size of PMN vesicles [110]. Furthermore, lipids seem to play an important role in PMN, as mutations in sphingolipid biosynthesis decreased PMN activity and V-ATPase exclusion under rapamycin treatment, similar as observed for ergosterol mutants [104]. In addition to protein key players, the electrochemical gradient across the vacuolar membrane was shown to promote invaginations of NVJs and induction of PMN [104]. While being excluded from the contact sites, the V-ATPase was still crucial for formation of invaginations and for scission of PMN-blebs. Here, the pumping activity rather than the presence of the V-ATPase was necessary for efficient PMN, as pharmacological V-ATPase inhibition blocked rapamycin-induced PMN.

Accumulating evidence indicates that the NVJs are involved in autophagic processes beyond PMN. The main tether Vac8 was demonstrated to contribute to the cytoplasm to vacuole targeting (Cvt) pathway, a selective form of autophagy with pure anabolic functions, via binding to Atg13 [182,183]. A change in the quaternary structure of Vac8, from arch-shaped when in contact with Nvj1 to a superhelical structure when associated with Atg13, selectively supported PMN or the Cvt pathway, respectively [184]. In addition, the NVJs might play a role in lipophagy, the selective autophagic degradation of LDs occurring at sterol-enriched vacuolar microdomains [34]. Specific receptors have not been identified so far [27,185,186], but lipophagy required not only the core autophagic machinery but also Vac8 [187]. While this hints at a possible contribution of NVJs to lipophagy, it might of course also reflect an MCS-independent function of Vac8. In mammalian cells, efficient lipophagy has been proposed to depend on the formation of ‘lipophagic junctions’, possible contact sites between LDs and autophagosomal membranes established by Rab GTPases, components of the autophagic machinery and additional adaptors [188].

In sum, while the precise contribution of NVJs and other MCS to lipophagy remains to be explored, the pivotal function of the contact sites between the nucleus/perinuclear ER and the vacuole for selective microautophagy of nuclear cargo in yeast is well established. Although autophagy of the nucleus (nucleophagy) is also described in mammalian cells [33,189], a possible role of MCS between the perinuclear ER and lysosomes in this selective form of autophagy remains to be investigated.

## 4. Conclusions and Outlook

**Figure 5:**
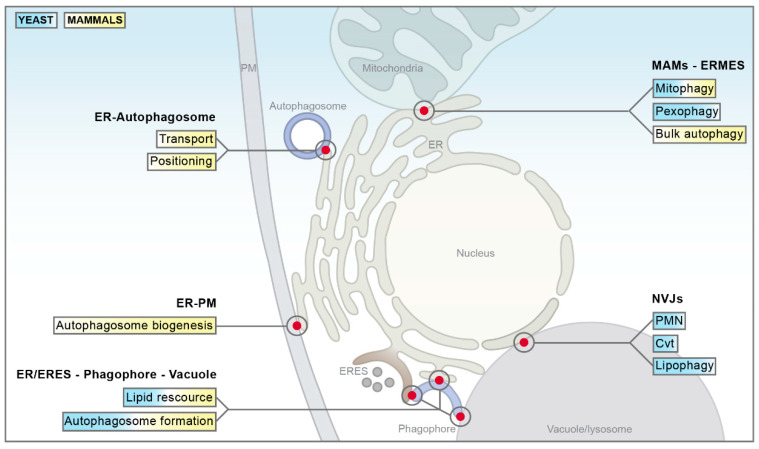
Summary of membrane contact sites involved in the regulation of autophagy. The main contribution of each interorganellar contact site to the autophagic process is shown in yeast (blue) and mammals (yellow). Please see main text for a detailed description. ER = endoplasmic reticulum; PM = plasma membrane; ERES = ER exit sites; MAMs = mitochondria-associated membranes; ERMES = ER–mitochondria encounter structure; NVJs = nucleus–vacuole junctions; PMN = piecemeal microautophagy of the nucleus; Cvt = cytoplasm to vacuole targeting.

The nexus between MCS and autophagy seems to be highly conserved across species boundaries (Figure 5). Though the notion that organellar apposition might be crucial for autophagic processes has been around for some time, we are just beginning to unravel the molecular details and functional diversity of MCS. Super-resolution microscopy and other techniques paved the way for the discovery of an increasing number of contact sites across the eukaryotic kingdom, thereby often establishing parallels between yeast and higher eukaryotic organisms [150]. Considering the high inter-species conservation of the autophagic process itself, it is not surprising that fundamentals of MCS-regulated autophagy are conserved. This includes but is not limited to ER–mitochondria contact sites, differing in their molecular architecture but central to mitophagy in both yeast and mammals, the contact sites between the expanding phagophore and the ER as well as the function of ERES as membrane resource for yeast and mammalian autophagosomes [150].

Additional contact sites might contribute to or directly participate in autophagic processes, for instance the MCS between the ER and trans-Golgi network. Considering that both organelles have direct implications in autophagy and that VAP proteins as the molecular determinants of these contacts govern several stages of this process [93,94,140,156,190], one might speculate that the proximity between these organelles indeed directly influences autophagy. Another potential candidate would be the vacuole and mitochondria patch (vCLAMP), the MCS between mitochondria and vacuoles in yeast [63,64], as Ypk9, the yeast orthologue of ATP13A2, a protein regulating autophagy in mammals [191], is enriched at vCLAMP [63,64]. As similar MCS between lysosomes and mitochondria exist in mammalian cells [192,193], further research to analyze potential implications of these MCS in autophagic processes will be important.

Although MCS are implicated in various autophagic processes, they seem to particularly contribute to phagophore expansion. While mechanistic details are still unexplored, MCS between the expanding phagophore and other organelles seem to influence the kinetics of autophagosomal biogenesis. Considering that the formation of a mature autophagosome is a fast process, estimated to take only about 10 min [194], the rapid mobilization of membrane sources for efficient phagophore expansion might be the rate-limiting step for autophagosomal biogenesis. While for instance single-membraned vesicles containing Atg9 serve as precursors for phagophore formation, this only contributes to the early steps of autophagosome formation [121]. The quantity of lipids delivered via this system is most likely insufficient to fulfil the high demand for membranes necessary for further autophagosomal membrane expansion downstream of phagophore nucleation. The identification of novel MCS directly connecting the phagophore and the ER, the central organelle for cellular lipid synthesis, provided an alternative and probably complementary route to deliver lipids to the expanding phagophore membrane [195]. This physical contact established by the conserved Atg2-Atg18 complex is suggested to facilitate rapid transfer of lipids and to efficiently drive phagophore expansion. Very recently, a mechanism for fast phospholipid supply to the phagophore has been proposed [195]. The authors could demonstrate that spatially-restricted de novo phospholipid synthesis at sites of close proximity between the ER and the nucleated phagophore drives the expansion of the phagophore membrane. Specifically, the targeting of the conserved acyl-CoA synthetase Faa1, which activates fatty acids, to nucleated phagophores at the ER was essential for fatty acid channeling into local phospholipid synthesis to produce lipid resources for phagophore expansion [195]. While it seems likely that these newly synthesized phospholipids are transported back to the phagophore membrane via non-vesicular transfer at physical contact sites between the ER and the phagophore—e.g., involving the Atg18-Atg2 tethering complex—the underlying mechanisms of this directional lipid flow remain elusive so far. A comprehensive characterization of autophagosomal membrane expansion kinetics in absence and presence of phagophore-emanating MCS is needed to provide further insights into their physiological relevance for autophagosome formation. It is reasonable to presume that any age-associated and/or disease-related decline of phagophore-emanating MCS will compromise lipid transfer to the growing autophagosomal membrane, thus slowing autophagosome formation. Given the importance of autophagy for cellular proteostasis and quality control [196], a deceleration of autophagosomal biogenesis will most probably entail deleterious consequences for cellular fitness and survival. Deciphering the precise role of MCS in autophagic processes will be an important task for future research. Taking advantage of the high inter-species conservation, the combined application of yeast and higher eukaryotic models will advance our understanding of the interplay between MCS and distinct autophagic processes and its impact on cellular homeostasis and survival.
